# The Generalized Cognitive Diagnosis Model Framework for Polytomous Attributes

**DOI:** 10.1017/psy.2024.16

**Published:** 2025-01-03

**Authors:** Jimmy de la Torre, Xuelan Qiu, Kevin Carl Santos

**Affiliations:** 1 The University of Hong Kong, Faculty of Education, Hong Kong; 2 Australian Catholic University, Faculty of Education and Arts, Brisbane, Australia; 3 University of the Philippines – Diliman, College of Education, Quezon City, Philippines

**Keywords:** cognitive diagnosis models, G-DINA, parameter estimation, polytomous attributes

## Abstract

For classroom teaching and learning, classifying students’ skills into more than two categories (e.g., no, basic, and advanced masteries) is more instructionally relevant. Such classifications require polytomous attributes, for which most existing cognitive diagnosis models (CDMs) are inapplicable. This paper proposes the *saturated polytomous cognitive diagnosis model* (sp-CDM), a general model that subsumes existing CDMs for polytomous attributes as special cases. The generalization is shown by mathematically illustrating the relationships between the proposed and existing CDMs. Moreover, algorithms to estimate the proposed model is proposed. A simulation study is conducted to evaluate the parameter recovery of the sp-CDM using the proposed estimation algorithms, as well as to illustrate the consequences of improperly fitting constrained or unnecessarily complex polytomous-attribute CDMs. A real-data example involving polytomous attributes is presented to demonstrate the practical utility of the proposed model.

## 1 Introduction

A wide variety of cognitive diagnosis models (CDMs) exist in the literature. Typically, these models were proposed for dichotomous attributes appropriate for determining, say, skill mastery or nonmastery. However, in many applications, classifying students into more than two categories is more instructionally relevant. Such classifications require polytomous attributes, where the attribute levels can be ordinal categories (e.g., no mastery, basic mastery, and advanced mastery). For example, the proportional reasoning (PR) assessment developed to measure the PR skills for middle school (equivalently, secondary) students (Tjoe & de la Torre, 2013, 2014) involves two three-level polytomous attributes, namely, (a) comparing and ordering of fractions, where level 0 represents nonmastery of the attribute, level 1 the ability to compare two fractions, and level 2 the ability to order three or more fractions and (b) constructing ratios and proportions, where level 0 represents nonmastery, level 1 the ability to construct a single ratio, and level 2 the ability to construct a proportion, which is made up of two ratios. Attribute levels can also be nominal categories representing different content domains. For example, level 1 represents the prerequisite skills (e.g., add and subtract) in the attribute hierarchy, whereas level 2 the advanced skills (e.g., multiply and divide). In general, any *M*-level polytomous attribute can be equivalently represented by 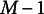 dichotomous attributes that follow a linear hierarchy (Leighton et al., 2004). In the example above, the polytomous attribute comparing/ordering fractions can be split into two dichotomous attributes, where the first deals with two fractions and the second with three or more fractions, and the mastery of the former is a prerequisite to the mastery the latter. The prerequisite relationship will constrain the number of possible mastery combinations to three, namely, 00, 10, and 11, which is equivalent to levels 0, 1, and 2 of the original polytomous attribute. Finally, it is important to underscore that this paper focuses on the attributes with more than two categories (i.e., polytomous *attributes*), rather than responses with more than two categories (i.e., polytomous *responses*). This distinction is necessary because both CDMs for polytomous attributes and those for polytomous responses have been referred to as polytomous CDMs in the literature.

To accommodate polytomous attributes, several CDMs have been developed in the literature, which are summarized and shown in Table 1 according to several key features such as general or constrained, link function, and core assumptions. For example, Templin (2004) extended the reparameterized unified model (RUM; Hartz, 2002) for polytomous attributes (RUM-PA) and proposed a constrained version (*c*RUM-PA), whereas Karelitz (2004) proposed the ordered category attribute coding (OCAC) framework in conjunction with the *deterministic input, noisy* “and” *gate* (DINA) model (Junker & Sijtsma, 2001) to define the mastery levels as multiple ordered categories. By defining accuracy with fast speed as the highest level of an attribute, accuracy with slow speed as intermediate level, and nonmastery as the lowest level, Wang and Chen (2020) extended the DINA model to be the response accuracy model (RAM) model to measure students’ fluency in answering the test items. Recently, Yakar et al. (2021) developed a fully additive model for polytomous attributes (*f*A-M), which accounts for the effects of each attribute levels. However, these models are deemed to be not general enough mainly because the models focused on a specific and constrained CDM.

**Table 1:** Summary of existing cognitive diagnosis models for polytomous attributes

Model	Framework	Link	Core assumptions
RUM-PA	Constrained	Log	Conjunctive
*c*RUM-PA	Constrained	Log	Conjunctive; equivalent distance between levels
OCAC-DINA	Constrained	Identity	Conjunctive
*f*A-M	Constrained	Identity	Additive
PDCM	General	Logit	Monotonic
*c*PDCM	Constrained	Logit	Monotonic; main effects of different levels are the same; interaction effects across levels are the same
GDM-PA	General	Logit	Depends on the choice of 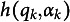
pG-DINA	General	Identity; logit; log	Monotonic; SALM
RAM	Constrained	Identity	Conjunctive; monotonic

*Note*: RUM-PA: reparameterized unified model for polytomous attributes; *c*RUM-PA: constrained RUM-PA; OCAC-DINA: *deterministic input, noisy* “and” *gate* model with ordered category attribute coding; *f*A-M: fully additive model for polytomous attributes; PDCM: polytomous diagnostic classification model; *c*PDCM: constrained PDCM; GDM-PA: general diagnostic model for polytomous attributes; pG-DINA: polytomous generalized DINA model; SALM: specific attribute level mastery; RAM: response accuracy model.

The existing general CDMs for polytomous attributes include those proposed within the log-linear cognitive diagnosis model (LCDM; Henson et al., 2009), that within the general diagnostic model (GDM; von Davier, 2008), and that within the generalized *deterministic input, noisy* “and” *gate* (G-DINA) model (de la Torre, 2011).

The polytomous diagnostic classification model (PDCM) framework (Bao, 2019) extends the measurement and structural models of the LCDM to the polytomous attribute setting. Note that in the PDCM framework, only the attribute patterns ( 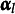 ) are polytomous, whereas the Q-matrix entries remain binary. In contrast, both the attribute pattern and Q-matrix entries are polytomous, for other CDMs in this paper, such as the OCAC framework, GDM, and the proposed framework. The probabilities between different levels in PDCM can be varied for greater flexibility or be equal for smaller number of parameters. The PDCM uses the dummy coding approach in which the *M* levels of an attribute are coded as 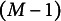 dummy variables and the combinations of the dummy variables—representing different knowledge states—are treated as the polytomous attribute levels. For example, for three levels of an attribute, they are coded with two dummy variables and as (0,0), (1,0), and (1,1) to represent nonmastery, intermediate mastery, and mastery. This coding approach might be workable when the number of levels in attributes and the number of attributes in a test are moderate. It becomes tedious and hard to interpret the representation of the knowledge states when the number of levels and attributes are large.

With a proper choice of the central component function, as in, the function 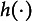 that maps the attribute levels using the Q-matrix entries, the GDM can flexibly accommodate polytomous attributes. For example, a useful and reasonable choice of 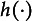 is defined as 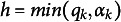 . As a result, an attribute level that is higher than 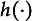 will not increase the probability of solving an item, whereas that is lower than 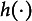 results in a lower success probability. In other words, while there is no distinction between groups who possess the required attribute level and who have an even higher level, there is distinction between groups whose attribute levels are lower than the required level. Nonetheless, the GDM for polytomous attributes (GDM-PA) has neither been examined with simulation studies in enough details nor applied to the real data.

With respect to the G-DINA model for polytomous attributes, namely, the pG-DINA model (Chen & de la Torre, 2013), the model relies on a core assumption, which is referred to the *specific attribute level mastery* (SALM), where each item is assumed to separate examinees into two reduced latent groups—those who are on or above a specific attribute level, and those who are below it. With the SALM assumption, some levels in the pG-DINA do not increase the success probability. Such a constraint may be too stringent because attribute vectors in the same reduced latent group are very likely to have varying levels with respect to the required attributes and thus their probabilities of success may not be identical.

The first and primary aim of this paper is to propose a general CDM framework for polytomous attributes, the *saturated polytomous cognitive diagnosis model* (sp-CDM), which is analogous to the G-DINA model for dichotomous attributes. Specifically, the proposed model extends the pG-DINA model by relaxing the SALM assumption and allows for the different attribute levels to contribute differentially to the success probability. This work also aims to derive the special cases of the sp-CDM under different constraints and show the mathematical relationships between the sp-CDM and the existing CDMs for polytomous attributes and its special cases. Third, this work aims to address the estimation of the sp-CDM, to examine parameter recovery using the proposed estimation algorithms, and the consequences of fitting constrained and unnecessarily complex models across a range of conditions. Finally, the study aims to demonstrate the application of the sp-CDM with a real data of PR assessment.

This paper contributes to the literature by developing a unified framework for polytomous attributes. The proposed model has three unique features: (1) Compared to the existing CDMs for polytomous attributes, where some attribute levels share identical success probabilities, the sp-CDM allows for different attribute levels to have their unique contributions to the success probability; (2) the sp-CDM is formulated with alternative link functions, thus, making it more general; and (3) due to the different model formulations, the existing models can be mathematically shown to be special cases of the various forms of the sp-CDM with appropriate constraints. Despite the similar structure of this work to that of the G-DINA (de la Torre, 2011) or the pG-DINA (Chen & de la Torre, 2013) model, the fundamental differences are substantial. Specifically, the formulations, the estimations, and the implications of three models are substantially different.

## 2 The generalized cognitive diagnosis model framework for polytomous attributes

The generalized CDM framework for polytomous attributes can be expressed as three saturated models under different link functions. Let *J* be the number of items, *K* the number of attributes, and 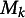 the number of levels of attribute *k*. For notational convenience, but without loss of generality, it can be assumed that 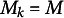 , indicating the number of levels is identical for all attributes. Thus, there will be a total of 
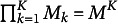
 attribute patterns or latent classes. Let 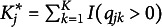 be the number of required attributes for the item *j*, *j*=1, …, *J*. Again, for notational convenience, let the first 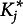 attributes be the required attributes for item *j*. We use 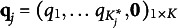 to denote the required levels in the 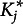 attributes to answer the item *j* correctly, and 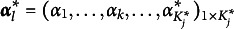 the *l*th reduced attribute pattern or latent group. As can be seen from above, the entries in both the 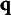 and 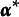 can have more than two categories.

To illustrate, consider 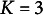 , 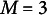 , and 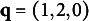 , which indicates that the first level of 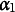 and the second level of 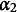 are required for the item, hence, 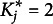 . For this example, there are 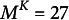 latent classes, which will be partitioned into 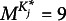 latent groups. Specifically, for this item, the latent classes 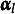 and 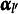 are classified in the same latent group when 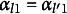 and 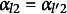 . For example, the latent classes 000, 001, and 002 all belong to the latent group 00.

The item response function (IRF) of the proposed model using the identity link function is given by 
(1)

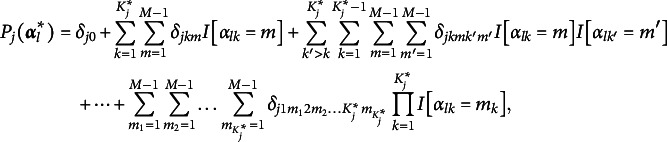

where 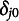 is the intercept, 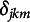 is the main effect of the *m*th level of attribute *k*, 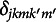 is the two-way interaction effect of the *m*th level of attribute *k* and the 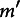 th level of attribute 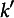 , and 
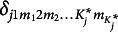
 is the 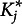 -way interaction effect of the 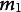 th level of the 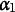 , 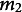 th level of the 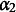 , up to the 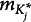 th level of 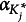 . It can be further noted that the subscript *m* of 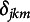 indicates that each attribute level in 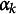 contributes differentially to the success probability, as in, the steps between adjacent levels vary (e.g., the step between “no mastery” and “basic mastery” is different from that between “basic mastery” and “advanced mastery”). To reduce the number of parameters and, hence, model complexity, it can be assumed that the steps between levels within 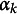 are identical, which reduces 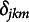 to 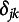 .

In addition to the identity link function, the sp-CDM can also be formulated with the logit and log links. Despite the similar forms, the models using different link functions are essentially different in terms of the values and interpretations of the parameters. For this reason, different notations are used for parameters under formulations with logit and log link functions.

For the logit link, 
(2)

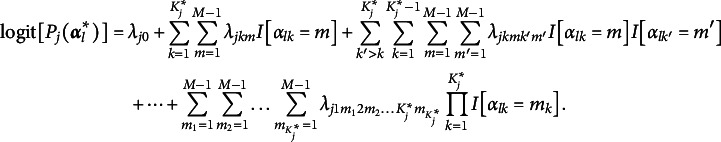



For the log link, 
(3)

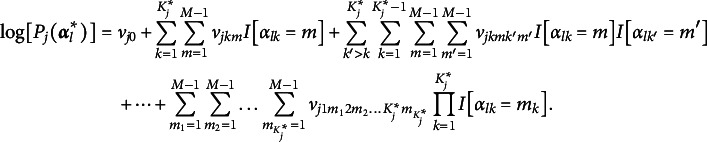



Equations (1), (2), and (3) are referred to as the *saturated polytomous cognitive diagnosis model* (sp-CDM) under the identity, logit, and log link functions, respectively. The number of parameters for item *j* for the three models is equal to the number of latent groups (i.e., 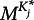 ). Thus, the models offer much greater generality compared to the existing CDMs for polytomous attributes. Although flexible, the large number of parameters in these models can make their estimation challenging. Therefore, simpler and more interpretable models with fewer parameters are sometimes warranted. Note that the number of parameters for the saturated models does not take into account the required attribute levels—it is computed as the product of the maximum levels of the required attributes. Thus, in the example above, in addition to 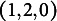 , the q-vectors 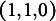 , 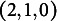 , and 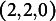 will result in the same saturated models.

## 3 Special cases

This section introduces several simplified CDMs for polytomous attributes with different assumptions, namely, the conjunctive, disjunctive, and additive assumptions, and how they can be derived from the sp-CDM by imposing appropriate constraints.

**The conjunctive version of the sp-CDM**

In the conjunctive version of the sp-CDM (conj-sp-CDM), it is assumed that examinees should possess levels that are equal to or higher than the required levels in *all* the required attributes are expected to answer the item correctly. Alternatively, persons who lack at least one of the required attributes, or possess levels lower than those required in at least one of the required attributes, are expected to answer the item incorrectly. Hence, the IRF of the conj-sp-CDM can be expressed as 
(4)

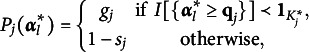

where the symbol 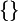 insides 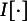 indicates that the operation be carried out attribute by attribute and 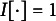 if 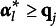 and 0 otherwise. 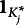 is a vector of ones and of length 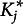 . The 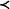 symbol indicates with respect to 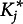 required attributes, which is a partially order set of *K* attributes, at least one of the elements in the results of 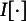 is less than 1. As shown in Equation (4), the conj-sp-CDM has two parameters for item *j*.

On the surface, the formulation of the conj-sp-CDM is similar to that of the DINA model. However, the parameters in Equation (4) require more complicated interpretations. For example, the 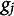 in the equation is the probability of correctly answering item *j* for individuals who lack at least one of the prescribed attributes, *or* who possess the required attributes, but with levels lower than required levels in the prescribed attributes; the 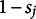 represents the probability of individuals who have attribute levels that are all at least equal to the required levels answering the item incorrectly. Thus, in the conj-sp-CDM model, the 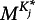 attribute vectors are classified into two latent groups—attribute vectors that jointly satisfy the required levels prescribed for item are classified in one group and the rest of the attribute vectors in the other group.

The conj-sp-CDM model can be derived from the identity sp-CDM (i.e., Equation (1)) by imposing the following three constraints:

(1) All the 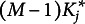 main effects are equal to zero, as in, 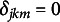 for 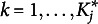 and 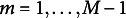 ;

(2) All the 
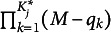
 interaction terms involving attribute levels at least equal to the required attribute levels are identical, as in, 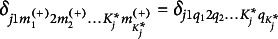 , where 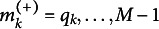 ; and

(3) The remaining 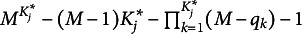 interaction effects involving at least one attribute level below the required level are all equal to zero.

With the conjunctive assumptions imposed on the identity sp-CDM, the IRF of the conj-sp-CDM can also be expressed as 
(5)

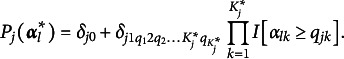



**The disjunctive version of the sp-CDM**

In the disjunctive version of the sp-CDM (disj-sp-CDM), the IRF is given by 
(6)

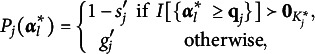

where 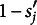 is the probability of not slipping for persons who possess levels that are equal to or higher than the required levels in at least one required attribute, and 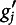 is the success probability that persons who possess none of required attributes, or possess at least one of the required attributes, but all of which have levels that are lower than the required levels. As such, the disj-sp-CDM has two parameters for each item.

The disj-sp-CDM can be derived from the identity sp-CDM with the following constraints:

(1) The main effects 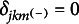 and 
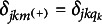
 , where 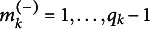 represents levels of attribute *k* that are lower than the required level 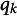 , and 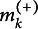 is as defined as above.

(3) The remaining interaction effects (i.e., those involving at least one 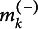 ) are of equal to zero.

With these assumptions, the identity sp-CDM can be reduced to be the disj-sp-CDM as 
(7)

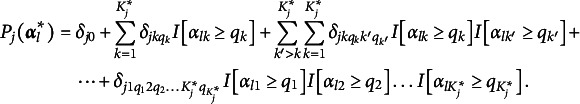



**The fully additive model for polytomous attributes**

The fully additive model for polytomous attributes (*f*A-M; Yakar et al., 2021) assumes that mastering level *m* of required attribute *k* increases the success probability on item *j* by 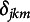 , and its contribution is independent of the contributions of the levels of the other attributes. By retaining only the main effects in Equation (1), the *f*A-M can be obtained, which has the following IRF: 
(8)

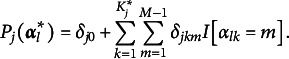



The *f*A-M has 
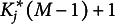
 parameters for item *j*. As with the saturated models, the number of parameters of the *f*A-M does not depend on the required levels, only the maximum levels of the required attributes. Using the required level 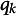 as a cutoff value, two simplified and interpretable models can be obtained from the *f*A-M. Specifically, in the first simplified *f*A-M, mastering an attribute level that is higher than 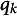 will contribute to higher probability of answering an item correctly, whereas mastering those that are lower than 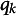 will have equal success probability. Hence, 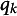 serves as a minimum bar and will be denoted as min-*f*A-M. The IRF for the min-*f*A-M can be expressed as 
(9)

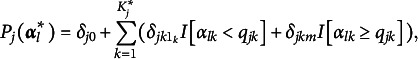

where 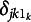 represents the main effect of 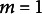 in attribute *k*. The number of parameters for item *j* in the min-*f*A-M reduces to 
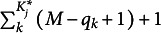
 .

In contrast to the min-*f*A-M, the second simplified *f*A-M assumes 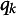 is a maximum requirement and will be denoted as max-*f*A-M. The model is similar to the GDM in that the success probabilities for attribute levels that are higher than 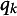 are equal to each other in both models, which equal to the probability of level 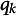 . However, those of levels that are lower than 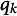 are different. The lower the level, the lower the success probability. The IRF for the max-*f*A-M can be expressed as 
(10)

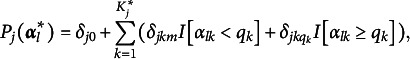

where 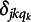 is the main effect of level 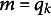 in attribute *k*. The max-*f*A-M has 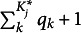 parameters for item *j*.

## 4 The connections between the sp-CDM and the existing models

**Figure 1:**
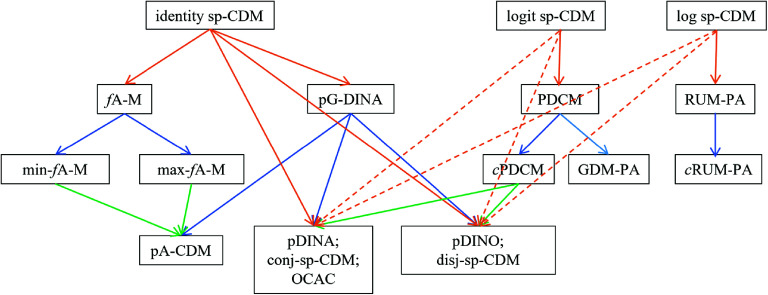
The generalized cognitive diagnosis model framework for polytomous attributes.Note: sp-CDM: saturated polytomous cognitive diagnosis model; *f*A-M: fully additive model for polytomous attributes; pG-DINA: generalized deterministic input, noisy “and” gate model for polytomous attributes with the specific attribute level mastery (SALM) assumption; PDCM: saturated polytomous-attribute diagnostic classification model; RUM-PA: reparameterized unified model for polytomous attributes; min-*f*A-M: *f*A-M using 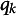 as a minimum requirement; max-*f*A-M: *f*A-M using 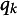 as a maximum requirement; *c*PDCM: constrained PDCM; GDM-PA: general diagnostic model for polytomous attributes; *c*RUM-PA: constrained RUM-PA; pA-CDM: additive model for polytomous attributes; pDINA: deterministic input, noisy “and” gate model for polytomous attributes; pDINO: deterministic input, noisy “or” gate model for polytomous attributes; conj-sp-CDM: conjunctive version of sp-CDM; disj-sp-CDM: disjunctive version of sp-CDM; OCAC: ordered category attribute coding framework. The colors orange, blue and green can be interpreted as the number of steps (i.e., 1, 2, and 3 steps) for the reduced models to be derived from the saturated model of a particular link function. For example, the pA-CDM can be derived from the identity sp-CDM through pG-DINA (two steps) or through *f*A-M then either min-*f*A-M or max-*f*A-M (three steps). The dashed lines indicate that the reduced models can also be shown to be special cases of sp-CDM with logit or log link functions.

This section shows, both mathematically and graphically, the connections between the sp-CDM and the existing models. Specifically, the existing CDMs for polytomous attributes can be formulated as special cases of the sp-CDM using different functions. A diagram (i.e., Figure 1) and a table (i.e., Table 2) help illustrate the connections between the sp-CDM and the existing models and those among the existing models.

**Table 2:** The relationships between sp-CDMs and the existing CDMs for polytomous attributes

Existing CDMs	Constraints on the sp-CDM	Special cases
pG-DINA	sp-CDM with 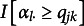	OCAC
OCAC	pG-DINA without interaction terms	—
*f*A-M	identity sp-CDM with main effects of levels	—
PDCM	logit sp-CDM	*c*PDCM; GDM-PA
GDM-PA	logit sp-CDM without interaction terms	—
RUM-PA	log sp-CDM with 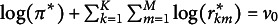 and 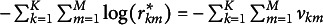	*c*RUM-PA

*Note*: sp-CDM: saturated polytomous cognitive diagnosis models; pG-DINA: generalized deterministic input, noisy “and” gate model for polytomous attributes with the specific attribute level mastery (SALM) assumption; OCAC: ordered category attribute coding framework; *f*A-M: fully additive model for polytomous attributes; PDCM: saturated polytomous-attribute diagnostic classification models; *c*PDCM: constrained PDCM; GDM-PA: general diagnostic model for polytomous attributes; RUM-PA: reparameterized unified model for polytomous attributes; *c*RUM-PA: constrained RUM-PA.

### 4.1 The identity model: pG-DINA and *f*A-M

The pG-DINA model can be obtained from the sp-CDM by replacing 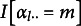 with 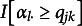 . With respect to the *f*A-M, as shown in Equation (8), it is the identity sp-CDM that retains the main effects only.

### 4.2 The logit model: PDCM and GDM-PA

In the PDCM framework (Bao, 2019), the logit of the success probably in answering item *j* correctly is given by 
(11)

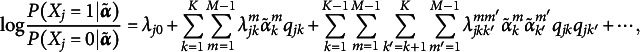

where 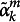 is the dummy variable for level *m* of attribute *k*, 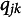 is equal to 1 if *k*th attribute is measured by item *j*. 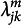 is the main effect of level *m* for attribute *k*, and 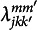 is the two-way interaction effect for level *m* of attribute *k* and level 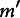 of attribute 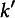 . As mentioned earlier, 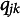 in the PDCM are still binary values. Hence, the PDCM is a special case of the logit sp-CDM.

In the GDM (von Davier, 2008), the IRF can be expressed as 
(12)

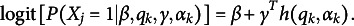

As mentioned earlier, for polytomous attributes with 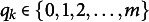 and 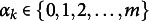 , a useful and reasonable choice of 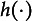 in GDM is defined as 
(13)

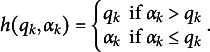

To this end, Equation (12) is equivalent to 
(14)

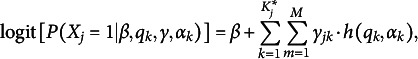

where 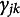 is the increase in the logit of the probability of success for every level of 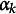 mastered up to the required level (i.e., 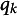 ).

### 4.3 The log model: RUM-PA

In the polytomous attribute RUM (Templin, 2004) with 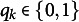 and 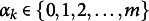 , the success probability is given by 
(15)

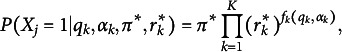

where 
(16)

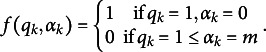

Like in the PDCM, the Q-matrices in the RUM-PA are assumed to be dichotomous, whereas the person attributes are assumed to be polytomous. Equation (15) can be written with respect to different attribute level *m* as 
(17)

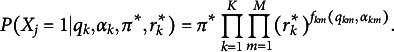

Equation (17) can be rewritten using 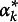 with 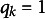 as 
(18)

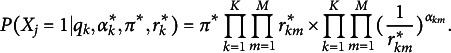

Thus, 
(19)

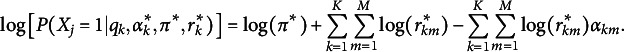

By setting 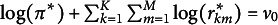 and 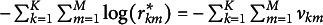 , Equation (19) is a special case of log sp-CDM without the interaction terms.

## 5 Model estimation

The estimation algorithms for the parameters, the corresponding standard errors (SEs), and the person attribute patterns in the sp-CDM model are primarily similar to those in estimating the parameters of the G-DINA model described in de la Torre (2011) and those of the DINA model in de la Torre (2009). Specifically, parameters for the saturated forms of the sp-CDM can be estimated via the marginal maximum likelihood estimation method with an expectation-maximization (MMLE/EM), and those of reduced models can be estimated by incorporating appropriate design matrix in the MMLE/EM procedure. Details can be found in Appendix A.

## 6 Simulation study

### 6.1 Design

Two research questions were investigated in the simulation study. First, how well can the parameters of the sp-CDM be recovered with the proposed estimation algorithms? And second, how does the fit of the sp-CDM compare with those of constrained and simplified models across different data generation assumptions. Due to time and space constraints, we focus on the identity sp-CDM and its two special cases, namely, the pG-DINA and 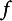 A-M, in this study. The details of the design are summarized in Table 3. The levels for the manipulated factors followed previous studies (e.g., de la Torre et al., 2021). In particular, the levels of item quality, defined as a function of the guessing and slip parameters, were computed as 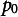 and ( 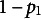 ). Specifically, items with ( 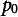 , 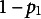 ) 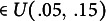 , 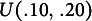 , and 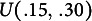 were classified as high, moderate, and low-quality items, respectively. To this end, the generating values for the intercept parameters were set to be .05, .10, and .15 under the three types of quality items. For the main effect parameters, the mean of the generating values are .16, .15, and .13, respectively, and for the interaction effect parameters, they are .1, .08, and .07, respectively. Attribute patterns were generated from a uniform distribution where all possible attribute patterns were equally likely. The Q-matrix for 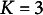 and 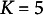 with 26 items is shown in Tables 4 and B.1, respectively, and the Q-matrix with 52 items is duplicate of respective Q-matrices. The Q-matrix in the simulation study was specified to satisfy the sufficient conditions similar to Theorem 4 in Fang et al. (2019). The GDINA package (Ma & de la Torre, 2019) and a customized program were used to generate the data and estimate the models. The monotonic constraints were imposed when estimating the models. A total of 168 conditions were examined, and each condition was replicated 100 times.

**Table 3:** Summary of the simulation design

Factor	Level
*M* (attribute levels)	3
*N* (sample size)	1,000, 2,000
*K* (number of attributes)	3, 5
*J* (number of items)	26, 52
Item quality	High, Moderate, Low
True/Fitted model	sp-CDM/sp-CDM
	sp-CDM/pG-DINA, sp-CDM/*f*A-M
	pG-DINA/sp-CDM, pG-DINA/pG-DINA,
	*f*A-M/sp-CDM, *f*A-M/*f*A-M

*Note*: sp-CDM: saturated polytomous cognitive diagnosis models; pG-DINA: generalized deterministic input, noisy “and” gate model for polytomous attributes with the specific attribute level mastery (SALM) assumption; *f*A-M: fully additive model for polytomous attributes.

**Table 4:** Q-matrix for conditions of three attributes in simulation study

Item	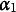	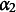	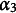	Item	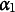	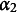	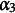	Item	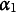	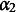	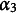
1	1	0	0	11	1	0	1	21	1	2	1
2	0	1	0	12	1	0	2	22	1	2	2
3	0	0	1	13	2	0	1	23	2	1	1
4	2	0	0	14	2	0	2	24	2	1	2
5	0	2	0	15	0	1	1	25	2	2	1
6	0	0	2	16	0	1	2	26	2	2	2
7	1	1	0	17	0	2	1				
8	1	2	0	18	0	2	2				
9	2	1	0	19	1	1	1				
10	2	2	0	20	1	1	2				

To answer the research questions, the simulation study was carried out in three steps. Step 1 was designed to answer the first research question. In this step, data under different conditions were generated following the sp-CDM (i.e., Equation (1)) and fitted with the true model (i.e., the sp-CDM). Steps 2 and 3 were designed to answer the second research question. In the second step, the pG-DINA model and 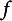 A-M were also fitted to the generated data in step 1 to investigate the consequences of fitting reduced models when neither the SALM nor the additive assumption holds. In step 3, two sets of data were generated following the pG-DINA model and 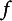 A-M, and fitted with sp-CDM, as well as their respective true model to investigate the consequences of fitting an unnecessarily complicated model (i.e., the sp-CDM) when the SALM or the additive assumption holds. For the sake of discussion, step 1 is referred to as the parameter recovery study, whereas steps 2 and 3 as the comparison study.

### Evaluation criteria

The dependent variables in the parameter recovery study were the bias and root mean square error (RMSE) of the estimated success probabilities of the reduced attribute patterns in item *j* (denoted as 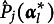 ), and were defined as 
(20)

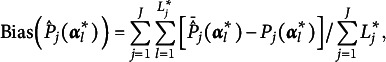

and 
(21)

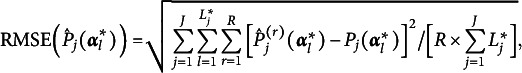

respectively, where 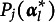 is the generating probability of 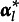 in item *j*, 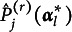 is the estimate of 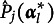 in the *r*th replication, 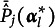 is the mean of 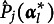 across *R* replications, and 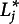 is the number of attribute patterns in item *j*.

In the comparison study, the dependent variables were the proportion of correctly classified attributes (PCA) and vectors (PCV), which were computed as 
(22)

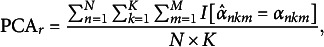

and 
(23)

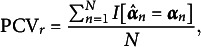

respectively, where 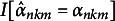 was used to evaluate the match between the estimated and generated attribute in the *r*th replication and 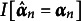 to attribute vectors. For both studies, the results are summarized using the average values of the variables across replications.

### Results

**Figure 2:**
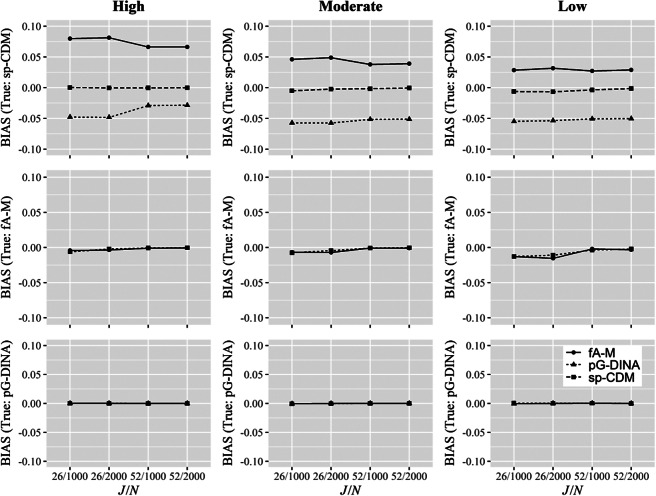
Bias in parameter recovery with three attributes.Note: sp-CDM: saturated polytomous cognitive diagnosis models; *f*A-M: fully additive model for polytomous attributes; pG-DINA: generalized deterministic input, noisy “and” gate model for polytomous attributes with the specific attribute level mastery (SALM) assumption. *J*: test length; *N*: sample size.

**Figure 3:**
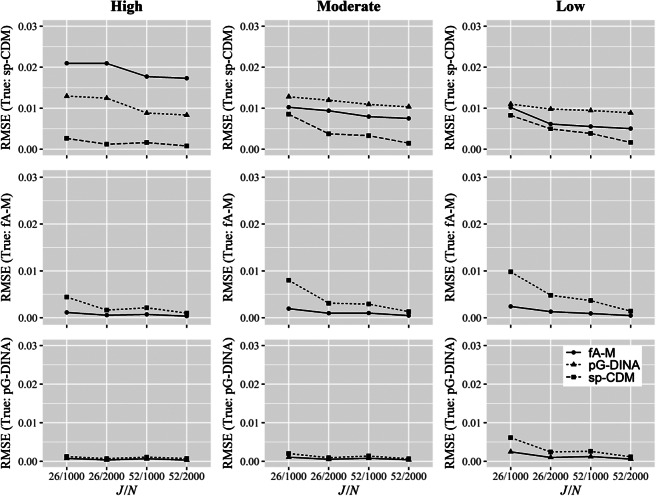
Root mean square error (RMSE) in parameter recovery with three attributes.*Note*: sp-CDM: saturated polytomous cognitive diagnosis models; *f*A-M: fully additive model for polytomous attributes; pG-DINA: generalized deterministic input, noisy “and” gate model for polytomous attributes with the specific attribute level mastery (SALM) assumption. *J*: test length; *N*: sample size.

For 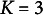 , the bias and RMSE under different conditions are shown in Figures 2 and 3, respectively, and PCA and PCV under the high, moderate, and low quality item conditions in Tables 5, 6, and 7, respectively. In particular, the upper panels of Figures 2 and 3 give the biases and RMSEs from fitting the sp-CDM to the sp-CDM data under different numbers of attributes, item qualities, test lengths, and sample sizes. The figures show that the parameters of the sp-CDM can be well recovered with the proposed estimation algorithms, particularly when high quality items were involved. For example, the mean biases were between 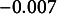 and 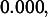 and the mean RMSEs were between 0.000 and 0.009 across all conditions. The upper panels of Tables 5, 6, and 7 reveal that the classification of attributes and vectors are satisfactory under the sp-CDM. For example, for the high quality item conditions (i.e., Table 5), the PCAs were between 88.2% and 96.9%, and the PCVs were between 71.9% and 89.3%.

**Table 5:** Correctly classified attributes (PCA) and vectors (PCV) (in %) with three attributes and high quality items

		Test length/sample size											
		26/1000			26/2000			52/1000			52/2000		
Gen	EC	1	2	3	1	2	3	1	2	3	1	2	3
sp-CDM	PCA1	90.6	86.3	87.2	91.0	86.2	87.3	96.6	91.7	95.4	96.9	91.6	95.4
	PCA2	88.9	84.3	82.9	89.4	84.5	82.7	95.7	90.8	92.4	96.1	91.1	92.8
	PCA3	88.2	82.5	82.2	88.8	82.6	82.6	95.4	89.7	92.2	95.7	90.2	92.4
	PCV	71.9	58.3	58.9	73.2	58.3	59.2	88.4	73.2	81.4	89.3	73.8	81.9
*f*A-M	PCA1	89.7	90.6	81.3	90.6	90.9	81.6	96.5	96.7	88.2	96.7	96.8	88.3
	PCA2	86.0	87.4	77.9	87.2	87.7	77.7	94.1	94.6	79.4	94.5	94.8	79.4
	PCA3	84.2	86.3	77.3	85.9	86.5	77.2	93.1	93.8	77.7	93.8	94.1	77.9
	PCV	65.4	69.1	47.4	68.5	69.7	47.3	84.8	86.2	51.8	86.0	86.6	52.0
pG-DINA	PCA1	96.8	95.1	97.0	97.0	95.2	97.1	99.5	99.0	99.5	99.5	99.1	99.6
	PCA2	96.1	94.2	96.3	96.3	94.3	96.4	99.3	98.9	99.4	99.3	98.9	99.4
	PCA3	95.5	93.4	95.6	95.6	93.5	95.7	99.1	98.5	99.2	99.1	98.6	99.2
	PCV	89.5	83.9	90.0	89.9	84.3	90.2	97.9	96.5	98.2	98.1	96.6	98.2

*Note*: Gen: Generating model; EC: Evaluation criteria; 1: sp-CDM; 2: *f*A-M; 3: pG-DINA; PCA*k*: PCA of attribute *k*.

**Table 6:** Correctly classified attributes (PCA) and vectors (PCV) (in %) with three attributes and moderate quality items

		Test length/sample size											
		26/1000			26/2000			52/1000			52/2000		
Gen	EC	1	2	3	1	2	3	1	2	3	1	2	3
sp-CDM	PCA1	79.1	78.2	75.9	79.9	78.4	76.2	89.3	87.7	83.2	90.2	87.8	83.2
	PCA2	76.5	75.5	73.4	77.4	76.2	74.1	87.1	85.5	79.6	88.3	86.0	80.2
	PCA3	75.0	74.7	74.0	76.7	75.0	74.4	86.6	84.9	81.3	87.9	85.0	81.2
	PCV	44.9	43.0	40.9	48.5	43.7	41.6	68.0	62.9	52.2	70.9	63.3	52.6
*f*A-M	PCA1	83.7	85.7	79.5	85.2	86.0	79.7	93.6	94.2	87.0	94.1	94.4	87.0
	PCA2	79.4	82.1	76.0	81.2	82.6	76.5	90.5	91.6	81.7	91.4	91.9	82.2
	PCA3	75.2	78.8	75.3	77.6	79.3	75.4	87.2	88.8	81.0	88.5	89.2	81.2
	PCV	50.4	56.4	45.1	54.5	57.2	45.7	74.4	77.3	56.3	76.7	78.0	56.7
pG-DINA	PCA1	92.5	90.0	92.9	92.6	89.9	92.8	97.9	96.7	98.1	98.1	96.8	98.1
	PCA2	91.5	88.4	92.0	91.8	88.7	92.1	97.6	96.3	97.8	97.8	96.3	97.9
	PCA3	90.6	87.1	91.1	91.1	87.7	91.4	97.1	95.6	97.3	97.4	95.8	97.5
	PCV	78.4	69.5	79.5	79.1	70.3	79.7	93.2	89.3	93.7	93.8	89.7	94.0

*Note*: Gen: Generating model; EC: Evaluation criteria; 1: sp-CDM; 2: *f*A-M; 3: pG-DINA; PCA*k*: PCA of attribute *k*.

**Table 7:** Correctly classified attributes (PCA) and vectors (PCV) (in %) with three attributes and low quality items

		Test length/sample size											
		26/1000			26/2000			52/1000			52/2000		
Gen	EC	1	2	3	1	2	3	1	2	3	1	2	3
sp-CDM	PCA1	73.2	71.8	72.1	74.0	73.9	72.3	84.2	83.6	79.8	85.4	83.9	80.1
	PCA2	70.1	69.2	68.8	71.2	70.8	69.9	81.2	80.8	76.1	82.6	81.5	76.7
	PCA3	67.1	66.0	66.2	67.9	67.0	67.8	77.3	77.2	74.1	79.2	77.3	74.2
	PCV	33.4	33.0	33.1	36.3	34.5	34.2	53.2	51.8	43.7	56.9	52.4	44.3
*f*A-M	PCA1	76.7	79.4	74.6	78.3	79.9	74.8	88.4	89.6	81.0	89.3	89.8	81.2
	PCA2	73.3	74.9	71.7	73.4	75.6	72.3	83.7	85.9	78.8	85.2	86.1	78.9
	PCA3	69.7	70.5	69.3	68.8	71.6	69.6	77.8	81.3	75.5	80.4	81.8	75.9
	PCV	43.0	43.1	37.5	40.2	44.2	37.9	58.2	63.6	47.6	62.0	64.3	48.0
pG-DINA	PCA1	80.6	73.8	82.4	82.0	74.3	82.9	90.9	83.7	91.9	91.6	83.9	92.1
	PCA2	80.5	75.3	82.2	81.9	76.4	82.8	91.0	86.4	91.8	91.7	86.9	92.1
	PCA3	80.6	74.5	82.4	82.1	75.1	83.0	91.1	84.6	91.9	91.6	84.1	92.0
	PCV	56.5	39.9	60.0	59.2	41.1	61.0	77.4	60.3	79.4	79.0	60.5	79.9

*Note*: Gen: Generating model; EC: Evaluation criteria; 1: sp-CDM; 2: *f*A-M; 3: pG-DINA; PCA*k*: PCA of attribute *k*.

In comparison, a closer inspection of the panels of Figures 2 and 3 and Tables 5, 6, and 7 reveals that fitting the reduced models, either the *f*A-M or the pG-DINA, to the sp-CDM data resulted in larger biases and RMSEs, or lower PCAs and PCVs, particularly when high quality items were used. Dramatically different results were obtained when the sp-CDM was fitted to the data generated using the reduced models, the biases, RMSEs, PCAs, and PCVs were similar to those obtained when the corresponding true models were fitted. These results can have important practical implications—when it is unclear which is the true model for an item, it is safer to fit the more general sp-CDM, rather than a particular reduced model.

**Figure B.1:**
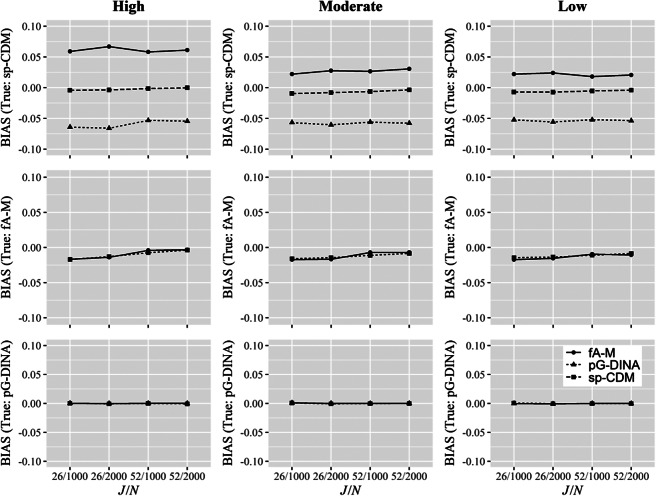
Bias in parameter recovery with five attributes.Note: sp-CDM: saturated polytomous cognitive diagnosis models; *f*A-M: fully additive model for polytomous attributes; pG-DINA: generalized deterministic input, noisy “and” gate model for polytomous attributes with the specific attribute level mastery (SALM) assumption. *J*: test length; *N*: sample size.

**Figure B.2:**
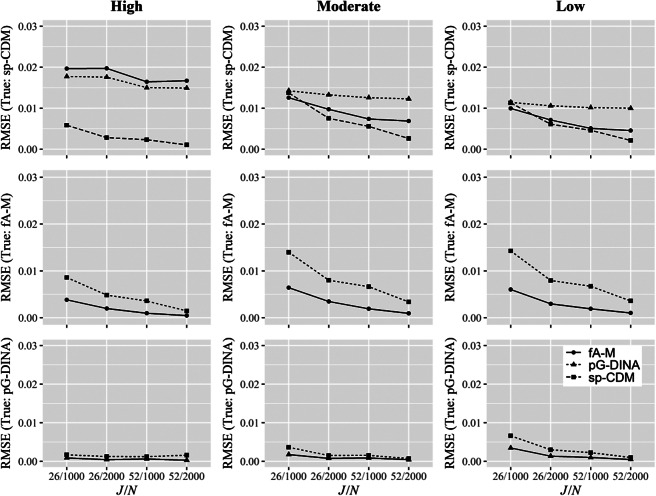
Room mean square error (RMSE) in parameter recovery with five attributes.Note: sp-CDM: saturated polytomous cognitive diagnosis models; *f*A-M: fully additive model for polytomous attributes; pG-DINA: generalized deterministic input, noisy “and” gate model for polytomous attributes with the specific attribute level mastery (SALM) assumption. *J*: test length; *N*: sample size.

Due to space constraints, the results for 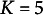 are given in Appendix B, where Figures B.1 and B.2 contain the bias and RMSE, respectively, and Tables B.2, B.3, and B.4 the PCA and PCV under the three item quality conditions, respectively. In general, the findings for 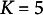 were similar to those for 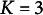 . However, it should be noted that for two conditions in Figure B.2 (i.e., 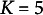 , 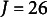 , 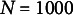 , and items are either of low or moderate quality), the sp-CDM produced larger RMSEs than *f*-AM even though the data were generated from the sp-CDM. This could be attributed to the instability of estimating the sp-CDM when a large number of parameters are involved, and the data are not sufficiently informative.

One reviewer noted that the RMSE results from both the 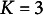 and 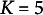 conditions for the sp-CDM and *f*AM tend to yield decreased RMSEs when tests contain more items and use smaller samples. For example, in the top panel of Figure 3, the RMSE results under the condition 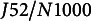 are smaller than those for the condition 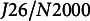 . To investigate this phenomena, an additional simulation was conducted where the dataset was generated from the sp-CDM with 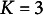 and high item quality for the two conditions (i.e., 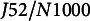 and 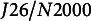 ). Three analyses were carried out as follows: In the first analysis, for the condition of 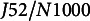 , the true attribute patterns for each person were assigned a posterior probability of 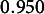 , and the remaining 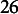 patterns for the person a probability of 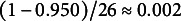 . In contrast, for the condition of 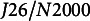 , the true attribute patterns for each person were assigned a posterior probability of 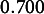 and other patterns a probability of 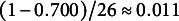 . This analysis mimics a scenario where the attribute patterns are better estimated in a smaller sample size condition than in a larger one. In the second analysis, the setting for the patterns’ posterior probabilities was reversed for the two conditions to mimic a scenario where the attribute patterns are better estimated in a larger sample size than in a smaller one. Finally, the patterns’ posterior probabilities were set using the results of the original simulation study. Specifically, the mean of the posterior probabilities for the true patterns across persons was computed for the two conditions—which are 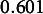 and 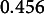 for the conditions of 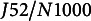 and 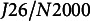 , respectively—and are used in the third analysis.

The biases and RMSEs for the additional simulation study are shown in Table 8. It was found that, for each analysis, the test that was assigned more accurate attribute pattern estimates resulted in better item parameter estimates (i.e., smaller bias and RMSE), even when the sample size was supposedly smaller. For example, in the first and third analyses, the biases and RMSEs under 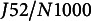 are smaller than those under 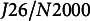 . These results demonstrate how more informative (i.e., longer) tests calibrated with a smaller sample size can produce better item parameter estimates.

**Table 8:** Additional simulation study: Bias and root mean square error (RMSE)

	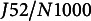		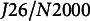	
Analysis	Bias	RMSE	Bias	RMSE
First	0.003	0.038	0.020	0.101
Second	0.020	0.103	0.003	0.029
Third	0.026	0.134	0.036	0.180

*Note*: In the first analysis, the posterior probabilities for the true attribute patterns are set to be 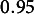 and 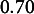 for the conditions of 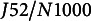 and 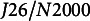 , respectively. In the second analysis, they are 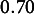 and 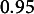 for the two conditions, respectively. In the third analysis, the probabilities are 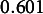 and 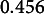 , respectively.

## 7 Real data example

### 7.1 Data and analysis

The responses in this example consisted of 1,408 middle school students in Hong Kong to a PR assessment described earlier. The assessment uses 31 multiple-choice items measuring six PR attributes, namely, ( 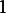 ) prerequisite skills and concepts required in PR, ( 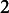 ) comparing and ordering fractions, ( 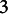 ) constructing ratios and proportions, ( 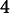 ) identifying a multiplicative relationship between sets of values, ( 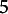 ) differentiating a proportional relationship from a non-proportional relationship, and ( 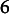 ) applying algorithms in solving PR problems, among which, the second and third attributes are polytomous with 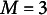 and other attributes are dichotomous. The Q-matrix for the empirical example is provided in Table 9, where each item requires one to four attributes (i.e., 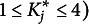 . This Q-matrix satisfies the identifiability conditions by Fang et al. (2019). The sp-CDM, *f*A-M, and pG-DINA model were fitted to the data. No monotonicity constraint was imposed in this analysis. The deviance, Akaike information criterion (AIC), and Bayesian information criterion (BIC) were used to compare the three fitted models.

**Table 9:** Q-matrix for the proportional reasoning data and the number of parameters under the sp-CDM, *f*AM, and pG-DINA model

Item	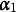	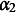	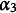	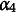	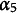	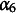	#Par			Item	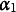	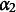	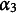	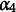	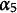	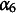	#Par		
							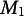	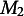	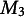								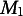	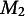	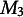
1	1	0	0	0	0	1	4	3	4	17	1	2	0	0	0	0	6	4	4
2	1	1	1	0	0	0	18	6	8	18	1	0	2	0	1	1	24	6	16
3	1	0	2	1	1	0	24	6	16	19	1	0	2	1	1	0	24	6	16
4	1	1	1	0	0	0	18	6	8	20	0	0	2	1	1	0	12	5	8
5	1	1	1	0	0	0	18	6	8	21	1	1	1	0	0	0	18	6	8
6	1	0	2	1	1	0	24	6	16	22	1	2	0	0	0	0	6	4	4
7	1	0	0	0	0	1	4	3	4	23	1	1	1	0	0	0	18	6	8
8	1	2	1	0	0	0	18	6	8	24	1	0	2	0	1	1	24	6	16
9	1	0	0	1	0	0	4	3	4	25	1	1	1	0	0	0	18	6	8
10	0	2	0	0	0	0	3	3	2	26	0	1	1	0	0	0	9	5	4
11	1	0	0	0	0	0	2	2	2	27	1	0	0	1	1	0	8	4	8
12	0	0	0	0	1	0	2	2	2	28	1	0	2	0	1	1	24	6	16
13	1	0	2	1	1	0	24	6	16	29	1	0	2	1	1	0	24	6	16
14	1	1	1	0	0	0	18	6	8	30	1	0	0	1	0	1	8	4	8
15	0	0	2	0	0	0	3	3	2	31	1	2	0	0	0	0	6	4	4
16	0	0	2	0	0	0	3	3	2	sum							416	148	254

*Note*: #Par: number of parameters; 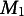 : sp-CDM, saturated generalized deterministic, input, noisy, “and” gate model for polytomous attributes; 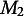 : *f*A-M, fully additive model for polytomous attributes; 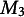 : pG-DINA, polytomous generalized DINA model.

### 7.2 Results

Table 10 shows the number of parameters and the fit statistics (i.e., deviance, AIC, and BIC) for the sp-CDM, *f*A-M, and pG-DINA models in the empirical example. All the fit statistics indicate that the sp-CDM fitted the data the best and the *f*A-M the worst.

**Table 10:** Fit statistics of the sp-CDM, *f*A-M, and pG-DINA model for the empirical example

	Number of parameters				
Fitted model	Items	Population	Deviance	AIC	BIC
sp-CDM	416	143	43360.17	44192.17	46376.13
*f*A-M	148	143	47983.06	48565.06	50092.79
pG-DINA	254	143	45524.30	46318.30	48402.52

*Note*: sp-CDM: saturated generalized deterministic, input, noisy, “and” gate model for polytomous attributes; *f*A-M: fully additive model for polytomous attributes; pG-DINA: polytomous generalized DINA model.

To quantify the discrepancies between the parameter estimates of item *j*, the root mean squared difference (RMSD) between any model pair is computed as follows: 
(24)

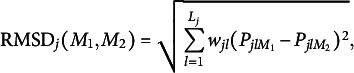

where 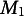 and 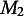 are pair of models, 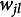 and 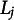 are the posterior probability of latent group *l* and the number of latent groups for item *j* based on the sp-CDM, respectively, and 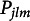 is the success probability of latent group *l* on item *j* based on a model *m*. Given in Table 11 are the results of RMSD for the 31 items, as well as the average RMSD for the entire test. On the average, *f*A-M and pGDINA model had the most similar the item parameter estimates (average RMSD = 0.171), whereas the sp-CDM and pG-DINA model had the most disparate estimates (average RMSD = 0.230). These results suggest that, at least for this empirical example, the two reduced model behaved more similarly to each other than they did to the saturated model. However, this pattern did not necessarily hold for all items. For example, the discrepancies between *f*A-M and pGDINA model turned out to be the largest for items 13 and 19.

**Table 11:** Root mean squared differences between the sp-CDM, *f*A-M, and pG-DINA model for the empirical example

Item	( 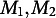 )	( 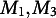 )	( 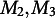 )	Item	( 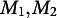 )	( 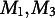 )	( 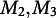 )
1	0.24	0.14	0.12	17	0.20	0.25	0.10
2	0.12	0.23	0.20	18	0.09	0.20	0.15
3	0.09	0.28	0.27	19	0.33	0.41	0.36
4	0.22	0.23	0.15	20	0.28	0.36	0.14
5	0.15	0.23	0.14	21	0.31	0.28	0.26
6	0.22	0.35	0.28	22	0.22	0.46	0.33
7	0.28	0.32	0.08	23	0.20	0.15	0.17
8	0.20	0.19	0.21	24	0.29	0.29	0.29
9	0.37	0.25	0.15	25	0.18	0.30	0.19
10	0.12	0.05	0.12	26	0.12	0.13	0.06
11	0.04	0.06	0.01	27	0.21	0.26	0.16
12	0.06	0.05	0.01	28	0.17	0.28	0.25
13	0.31	0.30	0.36	29	0.11	0.15	0.09
14	0.19	0.18	0.25	30	0.39	0.24	0.16
15	0.18	0.16	0.10	31	0.18	0.20	0.07
16	0.19	0.17	0.07	Average	0.20	0.23	0.17

*Note*: 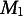 : sp-CDM, saturated generalized deterministic, input, noisy, “and” gate model for polytomous attributes; 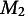 : *f*A-M, fully additive model for polytomous attributes; 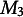 : pG-DINA, polytomous generalized DINA model.

**Figure 4:**
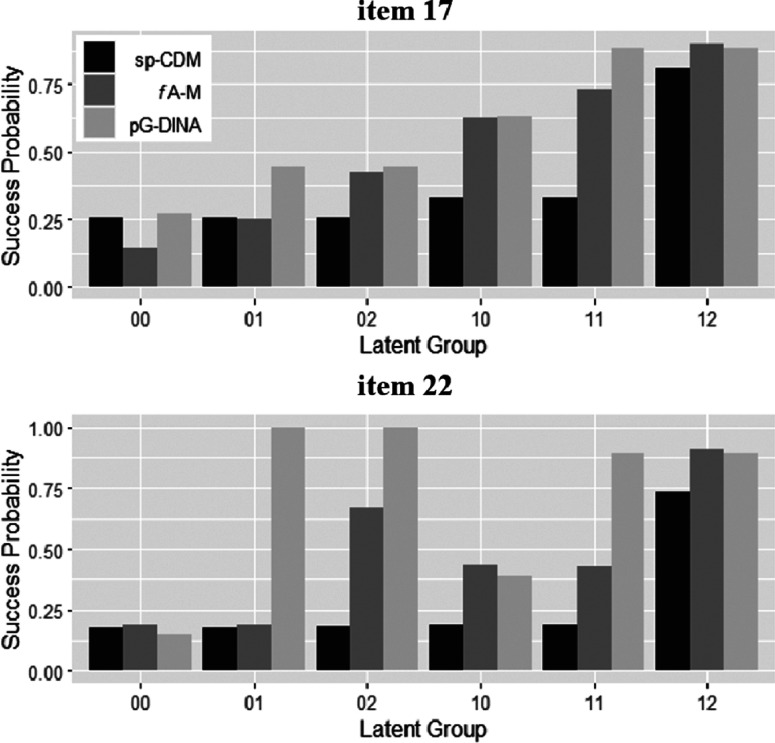
Success probabilities of latent groups in two items in the empirical example.Note: sp-CDM: saturated polytomous cognitive diagnosis models; *f*A-M: fully additive model for polytomous attributes; pG-DINA: generalized deterministic input, noisy, “and” gate model for polytomous attributes with the specific attribute level mastery (SALM) assumption.

To better understand how similar and disparate item parameter estimates look like, the side-by-side success probability bar graphs of two items requiring one dichotomous and one polytomous attributes are given in Figure 4. Item 17 had estimates that can be considered more similar, whereas item 22 more disparate. It should be noted that because RMSD is computed using latent group weights, large differences in the success probability estimates can have a limited impact on the RMSD, and vice versa. As can be seen from the upper panel, despite having more similar item parameter estimates, the success probabilities of item 17 for some latent groups (i.e., 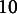 and 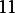 ) can be quite different. In contrast, the lower panel shows that item parameter estimates for item 22 were quite disparate, and huge discrepancies the success probabilities for latent groups for 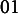 , 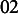 , and 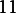 can be found, particularly, between sp-CDM and pG-DINA model.

The above comparisons were not meant to establish in general the similarities or differences between the three polytomous CDMs. Rather, it sought to better understand how the three models behave for a very particular empirical data set, which may provide insights into how future studies can be designed for the different models to be compared in a more systematic and comprehensive way.

The results can have important practical implications for diagnosing the mastery status of students. As shown in Figure 4, based on the sp-CDM, latent groups 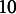 and 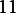 have slightly higher success probabilities on item 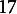 than latent groups 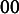 , 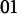 , and 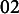 . This suggests that mastering the prerequisite skills and concepts increases the probability of getting item 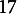 correctly; however, the success probability for this item is the highest when, in addition to mastering the prerequisite skills and concepts, an examinee also masters ordering fractions (level 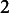 of 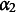 ). In comparison, again based on the sp-CDM, the estimated success probability on item 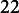 is the highest for latent group 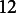 , whereas the success probabilities for other latent groups (i.e., 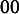 , 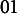 , 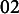 , 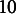 , and 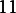 ) are similar to each other, indicating a conjunctive process for the item. Overall, an examinee should master levels 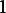 and 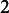 of the two required attributes, respectively, to optimize their success probabilities on these two items.

Of the possible 144 latent classes, 25 did not have any student. However, it should be noted that, although some latent classes had no observations, all the latent groups, from which estimates were derived, were nonempty, albeit some were small. For example, one of the 24 latent groups of items 3 had an expected size of 7.12. Of the remaining latent classes, 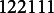 was the largest—about 35.5% of the students had this attribute pattern. Finally, the individual attribute prevalences were as follows: 88.2% mastered 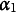 ; 16.2% and 68.5% were in levels 1 and 2 of 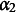 , respectively; 11.7% and 74.7% were in levels 1 and 2 of 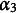 , respectively; and 75.4%, 70.5%, and 63.5% mastered 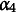 , 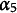 , and 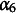 , respectively.

## 8 Discussion

Finer-grained feedback in the form of polytomous attributes can better inform classroom instruction and learning. However, the existing CDMs for polytomous attributes are deemed to be not general enough because most of them focused on a specific and constrained CDM or were proposed with very stringent assumptions. To this end, a more general framework, referred to as the sp-CDM, was proposed. The proposed model is a straightforward extension of the pG-DINA model (Chen & de la Torre, 2013), which itself is generalized from the G-DINA model for polytomous attributes, by relaxing its SALM assumption and can be formulated using the identity, logit, and log link functions. As such, the sp-CDM includes all the existing CDMs for polytomous attributes as its special cases. This paper has also illustrated the relationships between the sp-CDM and the existing CDMs mathematically and graphically.

In addition to the theoretical illustration, the estimation of the proposed model and the consequences of using constrained polytomous-attribute CDMs were examined via simulation study. The results showed that the parameter of the proposed model can be well recovered using the proposed estimation algorithms. On the other hand, improperly fitting a constrained polytomous-attribute CDM can lead to poor item parameter estimates and misdiagnosis of students’ true mastery levels while unnecessarily fitting the complex sp-CDM does little harm to the item and person estimation, particularly when high quality items were used. Moreover, the PR assessment example demonstrated the applicability of the proposed model to real data and its advantages over the constrained models.

Despite the promising results, this study is not without its limitations. First, although the simulation study manipulated several important factors, other relevant factors such as the number of attribute levels, the distribution of attribute patterns, and the link functions were fixed. Additional simulation studies are needed in the future to investigate the performance of the proposed model across a wider range of conditions. For example, the current simulation studies used the same number of attribute levels (i.e., three) across the items, which might not always the case in practice. Nevertheless, both the model and estimation algorithms proposed in this work are sufficiently general to apply to varying and a larger number of attribute levels, as evidenced by the empirical example. It would be interesting for future studies to extend the simulation design to incorporate attributes with more, as well as varying levels. This extension would provide useful information on how the increased attribute levels, which will lead to a greater number of latent classes, affects the sample size and test length required for the parameters of the proposed model to be estimated accurately. Moreover, this work focused on the uniform distributed attribute patterns. Future studies can extend the proposed model to other attribute distributions (e.g., higher-order distribution; de la Torre & Douglas, 2004), to understand the model performance across a wider variety of conditions.

Second, it has been recognized that failure to satisfy the Q-matrix identifiability conditions can result in poor parameter estimates. The existing necessary and sufficient conditions for the identifiability of CDMs in the literature focus on dichotomous attributes. (For example, see Chen et al. (2015), Chen et al. (2018), Chiu et al. (2009), DeCarlo (2011), Gu and Xu (2019), Liu et al. (2012), and Xu and Zhang (2016) for the conditions for the DINA model, and Fang et al. (2019), Gu and Xu (2021), Köhn and Chiu (2018), and Xu (2017) for general models.) In contrast, at present, only Fang et al. (2019) have discussed the identifiability conditions for the Q-matrix for polytomous attributes. However, the relevant results (i.e., Theorem 4) were limited to the sufficient conditions. To optimize the process of developing assessments that involve polytomous attributes, further research is needed to establish both the necessary and sufficient conditions specific to the identifiability of the sp-CDM and potentially its special cases.

Third, the current work focuses on polytomous attributes used in conjunction with dichotomous responses. Future research should extend the sp-CDM to also cover polytomous responses (e.g., Ma & de la Torre, 2020), as well as develop the associated estimation algorithms and computer program to implement such a model.

Fourth, although it has been noted that a polytomous attribute can be equivalently represented as a set of linearly structured dichotomous attributes, it is not clear to what extent the equivalence extends to methodologies that are specifically developed for each attribute type. For example, what modifications are needed for the empirical Q-matrix validation procedures developed for dichotomous attributes (e.g., de la Torre & Chiu, 2016) to be equivalent to Q-matrix validation procedures developed for polytomous attributes (e.g., de la Torre et al., 2021). Incidentally, a more general Q-matrix validation procedure that can be used with proposed model needs to be considered in future research.

Finally, this work proposes an MMLE/EM algorithm for estimating the sp-CDM and its special cases. The results of the simulation study demonstrate that the algorithm provides accurate estimates and is efficient in estimating the proposed models. However, challenges arise when the algorithm has to deal with the complexities associated with the sp-CDM in its most general form. For example, the estimation of standard errors in the saturated models becomes particularly challenging due to often encountered singular Hessian matrices.

**Figure B.3:**
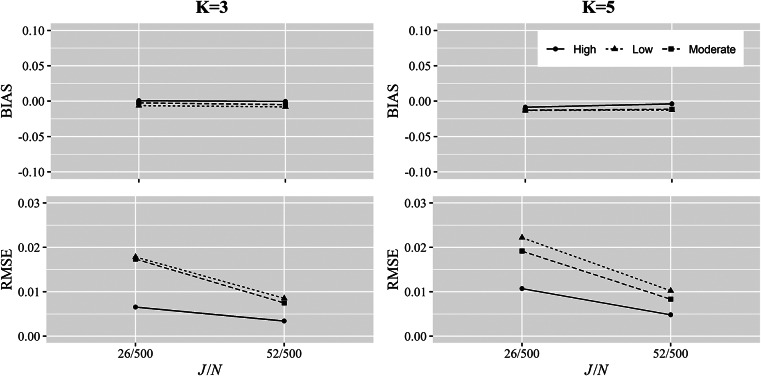
Results of parameter recovery for the sp-CDM with 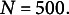 Note: sp-CDM: saturated polytomous cognitive diagnosis models; *J*: test length; *N*: sample size.

Furthermore, the parameter estimation becomes notably challenging in situations when the sample size is small relative to the number of attributes and attribute levels. To investigate this, an additional simulation was conducted with a sample size of 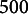 , maintaining the same settings as the primary simulation study. The results are given in Figure B.3 and Table B.5 in Appendix B. It was found that, under 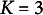 conditions, item parameter recovery and the PCA and PCV are satisfactory. However, for 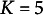 , although item parameter recovery and the PCA are only marginally acceptable, the PCV exhibits a significant deterioration, particularly when the item quality was low. The deterioration in the PCV performance may be attributed to the sparse latent classes—the expected number of individuals are 18 ( 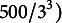 and two ( 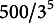 ) when 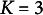 and 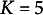 , respectively. These findings suggest that the sp-CDM may not be well suited for stand-alone small-sample settings (e.g., classroom assessment). Nonetheless, small-sample applications are still possible provided items can be calibrated a priori using a sufficiently large pool of individuals. In future research, it would be beneficial to explore the use of alternative estimation procedures such as nonparametric methods (e.g., Chiu et al., 2018) or Bayesian modal estimation (Ma & Jiang, 2021) to obtain robust person classification when polytomous attributes and small sizes are involved. It can be noted that small sample sizes impact not only the quality of item parameter estimates and attribute classification accuracy, but more so the standard error estimates. Thus, exploring various estimators of the CDM standard errors (Philipp et al., 2018) in the context of the proposed model need to be considered, particularly when the sample size is small.

## Item parameter estimation for the saturated polytomous cognitive diagnosis model via marginal maximum likelihood with an expectation-maximization algorithm

This appendix provides details of item parameter estimation in the sp-CDM. Due to space constraints, it focuses on the identity sp-CDM in particular and the algorithms are ready to be implemented to the sp-CDM with the logit and log link functions.

Based on the IRF of the saturated identity sp-CDM (i.e., Equation (1)) and assuming local independence, the marginalized likelihood of the data, denoted as  , is given by

(A1)

where  is the marginalized likelihood of the response vector of examinee *n*,  is the joint probability of the examinee’s response vector  conditional on attribute vectors  and the item parameters  , and  is the prior probability of the attribute vectors  .

The logarithm of  is

(A2)

To find the marginal likelihood equation for  of item *j*, take

(A3)

Then,

(A4)

Assuming local independence, the term  in Equation (A4) is given by

(A5)

where  is defined in Equation (1),  ,  for correct answer of examinee *n* in item *j* and  otherwise.

Hence, the derivative of  becomes

(A6)

where the product is over  rather than *j* because the derivation is with respect to  .

The second term in the right hand side of Equation of A6 is

(A7)

By substituting Equation (A7) to the right hand side of Equation (A6) and rearranging yields

(A8)

Substituting Equation (A8) to Equation (A4) yields

(A9)

where  is the posterior probability that examinee *n* is in the latent group  ,  is the number of examinees in the latent group  expected to answer the item *j* correctly, and  is the number of examinees expected to be in the latent group  .

Therefore, the marginal likelihood Equation (A3) can be written as follows:

(A10)

Solving Equation (A10) yields the MML estimation of  , which can be expressed as

(A11)

To convert the estimates of  into the item parameters  , the design matrix  is needed. With  , the estimates of  ,  ,  ,  ,  ,  can be computed as

(A12)

where  . This is identical to the design matrix used with G-DINA model (de la Torre, 2011). Due to the important role the design matrix  plays in the estimation of the sp-CDM, the following shows how  can be constructed.

Under the sp-CDM, the dimension size of design matrix is  , where  is the number of the parameters of the model of interest, which is  when converting  to  . To illustrate, let  and  for item *j*. This item has one intercept parameter, four main effect parameters for each level of the two nonzero levels of the required attributes, and four two-way interaction parameters between the levels of the required attributes, leading to a total of nine parameters. Hence,  in this example. The corresponding saturated design matrix is

(A13)

where rows 1 through 9 of  correspond to the latent groups  ,  ,  ,  ,  ,  ,  ,  , and  , respectively; columns 1 through 9 represent the intercept followed by the four main effects, and then by the four two-way interaction effect. For example, the success probability of the latent group  (i.e., row 6) is  , whereas that of the latent group  (i.e., row 9) is  .

The implementation of the MMLE/EM algorithm is as follows:

Step 1: The expectation (E) step

(1) Use the Equation (A5) and provisional item parameter estimates to compute the likelihood of each examinee’s response vector at each of the *L* attribute patterns.

(2) Use the Equation (A1) to compute the likelihood of the whole data. For convenience, the uniform distribution is usually chosen to initialize the prior distribution, as in,  in the first iteration. In subsequent iterations, the prior distribution is updated by replacing it with the posterior distribution, which itself is updated at the end of each iteration.

It is not uncommon that many examinees can have the same response vectors to the *J* items in a data set and the computations of  are often replicated. To increase the computational efficiency, an alternative way to compute the likelihood of data starts from grouping the *N* examinees’ response vectors into  possible response patterns. Let the item response patterns are denoted as  (  ) and the number of examinees possessing response pattern *s* is given by  . The likelihood of data can be calculated as  .

(3) Count the number of examinees expected to be in the latent group  among  latent groups and the number of examinees in the latent group  expected to answer the item *j* correctly and use them as the values of  and  in Equation (A9), respectively.

Step 2: The maximization (M) step

Solve the likelihood Equation (A3) using the values of  and  . Because these values depend on  ), which in turn, depends on the unknown item parameters, the likelihood equations are implicit and must be solved with an iterative procedure(e.g., Newton-Raphson procedure).

The E step and M step will be repeated unless certain criteria are met (e.g., the change of likelihood between two successive cycles is less than 0.001, the maximum number of iterations, say, 100 is reached).

## Q-matrix with five attributes and additional results in simulation study

**Table B.1:** Q-matrix for conditions of five attributes in simulation study

Item						Item						Item					
1	1	0	0	0	0	11	1	1	0	0	0	21	0	0	1	2	1
2	0	1	0	0	0	12	1	2	0	0	0	22	0	0	1	2	2
3	0	0	1	0	0	13	2	1	0	0	0	23	0	0	2	1	1
4	0	0	0	1	0	14	2	2	0	0	0	24	0	0	2	1	2
5	0	0	0	0	1	15	1	0	1	0	0	25	0	0	2	2	1
6	2	0	0	0	0	16	1	0	2	0	0	26	0	0	2	2	2
7	0	2	0	0	0	17	2	0	1	0	0						
8	0	0	2	0	0	18	2	0	2	0	0						
9	0	0	0	2	0	19	0	0	1	1	1						
10	0	0	0	0	2	20	0	0	1	1	2

**Table B.2:** Correctly classified attributes (PCA) and vectors (PCV) (in %) with five attributes and high quality items

		Test length/sample size											
		26/1000			26/2000			52/1000			52/2000		
Gen	EC	1	2	3	1	2	3	1	2	3	1	2	3
sp-CDM	PCA1	86.8	84.1	79.6	87.3	84.4	79.7	94.7	92.2	86.2	94.8	92.0	86.4
	PCA2	80.3	79.1	76.8	81.1	79.9	76.7	90.0	88.2	80.6	90.4	88.1	80.4
	PCA3	87.6	84.8	84.3	88.5	84.9	84.1	95.4	92.8	92.9	95.7	92.7	93.0
	PCA4	78.8	75.6	77.8	80.2	75.6	78.1	88.6	84.4	82.0	89.5	84.0	81.5
	PCA5	77.8	73.2	77.5	79.3	72.8	77.6	87.5	81.3	80.9	88.6	81.4	80.5
	PCV	38.1	30.0	30.7	40.7	30.4	30.8	63.6	50.8	41.6	65.6	50.1	41.1
*f*A-M	PCA1	87.6	88.0	79.8	88.2	88.3	79.2	94.9	95.0	82.2	95.2	95.3	82.4
	PCA2	80.9	81.1	79.0	81.4	81.8	78.9	89.6	89.8	79.9	90.0	90.0	80.0
	PCA3	84.4	86.1	79.9	85.6	86.7	80.0	93.1	94.0	84.4	93.8	94.1	84.4
	PCA4	77.4	79.9	78.7	78.1	80.3	78.3	85.9	88.8	78.1	87.9	89.0	77.9
	PCA5	78.2	80.8	77.7	79.8	81.5	77.7	87.4	89.8	78.5	89.1	89.9	78.3
	PCV	36.1	39.8	30.0	38.5	41.3	29.8	59.3	64.3	32.3	63.2	65.0	32.4
pG-DINA	PCA1	95.8	95.1	95.9	95.8	95.2	96.0	99.3	99.1	99.3	99.3	99.2	99.4
	PCA2	92.5	92.3	92.9	92.8	92.6	93.1	97.7	97.6	97.8	97.7	97.7	97.8
	PCA3	94.5	93.3	94.9	94.7	93.2	95.0	98.7	98.0	98.8	98.7	98.0	98.9
	PCA4	92.8	84.6	93.3	93.2	83.9	93.5	97.0	96.2	97.2	97.0	96.2	97.2
	PCA5	92.8	79.6	93.3	93.1	77.4	93.5	96.9	95.9	97.1	97.1	96.3	97.3
	PCV	73.1	54.7	74.6	73.9	52.8	75.0	90.1	87.5	90.7	90.3	88.0	91.0

*Note*: Gen: Generating model; EC: Evaluation criteria; 1: sp-CDM; 2: *f*A-M; 3: pG-DINA; PCA*k*: PCA of attribute *k*.

**Table B.3:** Correctly classified attributes (PCA) and vectors (PCV) (in %) with five attributes and moderate quality items

		Test length/sample size											
		26/1000			26/2000			52/1000			52/2000		
Gen	EC	1	2	3	1	2	3	1	2	3	1	2	3
sp-CDM	PCA1	75.1	75.0	73.4	76.3	76.0	73.3	86.2	85.8	79.5	86.8	85.9	79.2
	PCA2	69.5	67.3	70.0	70.4	67.2	70.1	79.1	77.9	75.2	80.5	78.2	75.3
	PCA3	72.9	74.3	73.4	74.5	75.0	73.5	84.6	84.7	81.1	86.1	84.9	81.0
	PCA4	68.1	69.5	70.1	69.1	69.3	70.3	80.6	79.8	76.4	80.3	79.9	76.6
	PCA5	67.4	67.3	69.6	68.5	67.3	70.1	76.7	76.5	75.5	78.4	76.7	75.3
	PCV	18.5	17.1	17.5	19.2	17.3	18.8	35.1	33.9	27.1	38.5	34.2	27.0
*f*A-M	PCA1	78.8	80.0	75.1	80.2	80.8	75.2	89.0	89.5	78.5	89.7	89.9	78.5
	PCA2	73.0	73.3	73.2	73.8	74.0	73.5	83.0	83.7	77.8	83.9	84.2	77.7
	PCA3	76.2	79.5	75.7	78.2	80.3	75.8	87.2	89.6	82.2	88.7	89.7	82.3
	PCA4	68.5	71.5	72.3	69.7	72.5	72.4	76.9	81.2	74.9	79.2	82.2	74.8
	PCA5	69.0	71.5	72.4	69.4	72.3	72.4	76.5	80.6	74.4	78.5	81.6	74.2
	PCV	20.7	24.2	21.6	22.4	25.5	21.9	37.8	44.3	27.0	41.6	45.9	27.1
pG-DINA	PCA1	91.1	90.2	91.3	91.4	90.5	91.5	97.6	96.9	97.7	97.7	96.8	97.7
	PCA2	85.8	85.3	86.2	86.1	85.5	86.5	94.3	94.0	94.4	94.4	94.0	94.5
	PCA3	88.0	85.5	88.8	88.5	85.5	89.0	95.9	94.2	96.2	96.1	94.4	96.3
	PCA4	84.5	74.6	85.8	86.0	73.8	86.5	92.0	90.8	92.5	92.4	91.0	92.7
	PCA5	85.0	73.3	86.1	86.3	71.6	86.9	92.0	88.5	92.5	92.3	87.9	92.6
	PCV	51.3	35.8	53.7	53.7	34.7	55.0	75.5	69.2	76.7	76.4	68.9	77.0

*Note*: Gen: Generating model; EC: Evaluation criteria; 1: sp-CDM; 2: *f*A-M; 3: pG-DINA; PCA*k*: PCA of attribute *k*.

**Table B.4:** Correctly classified attributes (PCA) and vectors (PCV) (in %) with five attributes and low quality items

		Test length/sample size											
		26/1000			26/2000			52/1000			52/2000		
Gen	EC	1	2	3	1	2	3	1	2	3	1	2	3
sp-CDM	PCA1	72.8	72.7	71.7	74.1	73.7	71.8	84.2	83.6	78.5	84.7	83.8	78.3
	PCA2	67.9	65.0	68.2	68.9	65.2	68.2	76.9	75.5	73.5	77.9	75.9	73.5
	PCA3	70.4	71.6	71.2	71.8	72.3	71.5	82.0	82.3	79.0	83.4	82.6	79.1
	PCA4	65.5	66.8	67.9	66.8	67.4	68.2	78.6	77.0	74.6	77.1	77.5	74.5
	PCA5	65.2	65.5	67.3	66.1	65.5	67.9	75.7	75.6	73.8	76.1	76.2	74.0
	PCV	16.1	14.5	16.0	16.6	15.0	16.4	31.0	30.1	24.6	32.9	30.7	24.6
*f*A-M	PCA1	74.2	75.7	72.5	75.5	76.4	72.6	85.4	86.1	78.9	86.1	86.4	79.0
	PCA2	66.9	69.5	68.1	68.0	68.0	68.3	75.9	76.7	73.8	77.2	77.7	74.0
	PCA3	69.3	73.0	70.4	71.0	73.8	70.9	80.5	83.7	77.7	82.3	84.0	77.8
	PCA4	64.3	67.4	67.3	65.7	68.2	67.6	72.6	76.3	72.8	74.4	77.1	72.9
	PCA5	62.1	68.6	66.5	64.0	67.1	67.1	69.7	73.7	70.7	71.3	74.3	70.8
	PCV	13.8	16.8	15.8	15.4	17.9	16.1	26.4	31.6	23.2	29.3	32.8	23.2
pG-DINA	PCA1	80.2	77.2	80.8	80.7	77.4	81.0	90.2	87.2	90.5	90.7	87.5	90.8
	PCA2	76.5	70.5	77.0	76.8	70.0	77.2	85.5	82.6	85.8	85.8	82.7	85.9
	PCA3	77.7	75.4	79.3	79.0	75.4	79.9	88.8	85.6	89.6	89.4	85.7	89.8
	PCA4	72.5	69.7	74.9	74.2	69.2	75.7	83.0	81.4	84.0	83.7	81.7	84.4
	PCA5	73.0	68.2	75.2	74.5	67.5	75.8	83.0	78.2	84.0	83.8	76.8	84.3
	PCV	27.9	19.1	30.6	30.0	18.6	31.7	49.6	39.4	51.6	51.3	39.0	52.4

*Note*: Gen: Generating model; EC: Evaluation criteria; 1: sp-CDM; 2: *f*A-M; 3: pG-DINA; PCA*k*: PCA of attribute *k*.

**Table B.5:** Correctly classified attributes (PCA) and vectors (PCV) (in %) for the sp-CDM with

	K=3								K=5						
	26/500				52/500				26/500				52/500		
EC	H	M	L		H	M	L		H	M	L		H	M	L
PCA1	89.3	76.0	70.1		96.1	87.8	82.0		85.7	73.5	71.2		94.2	84.8	82.7
PCA2	87.6	73.2	66.9		95.3	84.0	77.7		79.1	68.6	67.1		89.4	78.0	75.8
PCA3	86.8	72.5	64.2		94.9	84.6	74.4		86.6	70.7	69.2		94.7	82.0	79.8
PCA4	–	–	–		–	–	–		77.8	66.9	63.5		87.2	76.7	73.7
PCA5	–	–	–		–	–	–		76.6	66.2	63.2		86.2	75.5	72.6
PCV	68.5	40.6	30.1		87.2	62.8	47.5		35.2	15.7	13.4		60.1	31.4	26.7

*Note*: sp-CDM: saturated polytomous cognitive diagnosis models; EC: Evaluation criteria; H: High quality items; M: Moderate quality items; L: Low quality items; PCA*k*: PCA of attribute *k*.
